# Gender Distribution Among Invited Speakers at the Italian Orthodontic Society (SIDO) Conferences Held Between 2013 and 2023: A Cross‐Sectional Analysis

**DOI:** 10.1111/ocr.70001

**Published:** 2025-06-27

**Authors:** Valentina Lanteri, Alessandro Bruni, Andrea Abate, Cinzia Maspero, Elis Kola, Alessandro Ugolini

**Affiliations:** ^1^ Surgical, Medical and Dental Department of Morphological Sciences Related to Transplant, Oncology and Regenerative Medicine University of Modena and Reggio Emilia Modena Italy; ^2^ Department of Sciences Integrated Surgical and Diagnostic University of Genova Genova Italy; ^3^ Department of Biomedical Surgical and Dental Sciences University of Milan, Fondazione IRCCS Cà Granda, Ospedale Maggiore Policlinico Milan Italy

## Abstract

Scientific conferences play a key role in shaping professional visibility and recognition. Investigating gender balance among invited speakers can provide insight into broader patterns of inclusion in the context of orthodontic congresses. The purpose of this study was to evaluate gender representation among invited speakers at the Italian Orthodontic Society (SIDO) congresses from 2013 to 2023, identifying trends and potential disparities during this decade‐long period. A cross‐sectional review of the abstract books and programmes of the SIDO *Spring* and *Winter* congresses held between 2013 and 2023 was conducted. The gender, country of origin, affiliation, topic of presentation and H‐index were recorded for each invited speakers. Presentations lasting less than 30 min, involving more than three speakers, focusing on non‐orthodontic topics (e.g., dental hygiene or dental technician presentations) or held during sponsored sessions, roundtables and collateral events were excluded. Descriptive statistics were performed to summarise speaker characteristics. Univariate and multivariate logistic regression analyses were conducted to examine the effect of predictor variables, including year of presentation, country (non‐Italian speakers), H‐index (≥ 8) and affiliation (non‐academic) on the likelihood of a speaker being female. The Mantel–Haenszel test for homogeneity of odds ratios (OR) was used to assess whether trends in female representation were consistent over time. The predefined level of statistical significance was set at *p* < 0.05 (two‐sided). The analysis revealed a significant gender imbalance, with male speakers accounting for 77.2% and female speakers 22.8% throughout the study period (*p* < 0.001). Logistic regression indicated that the likelihood of a speaker being female increased by 6% for each additional year (OR: 1.055, 95% CI: 1.010–1.110, *p* = 0.015), while other variables such as country of origin, H‐index and affiliation were not significant. The majority of speakers were from Italy (62.3%), followed by the United States (9.6%) and Spain (3.2%). The topics discussed most frequently were Interdisciplinary (14.2%), New Technologies (8.4%) and Clear Aligner Treatment (8.2%). A large proportion of the invited speakers lacked academic affiliation (36.4%). Although the proportion of female speakers at SIDO congresses remains limited, the data reveal a steady annual increase in female representation. Nevertheless, a substantial gender imbalance persists, indicating that initiatives aimed at improving diversity could contribute to a more balanced representation in the future.

## 1 Introduction

Gender balance in academic conferences has been a topic of growing interest, with ongoing discussions about the distribution of male and female speakers. Being chosen as a speaker often indicates a significant level of expertise and represents a crucial step in career advancement within many scientific disciplines. Prior research has demonstrated that women are frequently underrepresented as speakers across several fields within science, technology, engineering and mathematics (STEM) [1, 2]. This underrepresentation aligns with broader academic dynamics, as female scientists have historically faced gender‐based bias, even amid efforts to address the contributing factors [3].

Studies in the medical field further emphasise a significant disparity between male and female scientists, particularly in research output and the associated advancement to senior academic roles or leadership positions [4, 5]. This imbalance is also reflected in governance roles within scientific publishing, with female participation in editorial or advisory boards of oral health journals ranging from 7% to 40%, and orthodontic journals reflecting an even lower figure, around 12% [6].

These visibility gaps persist despite the growing number of women in the dental profession. According to 2024 data (Supporting Information), women represent approximately 29.3% of all registered dentists in Italy. However, this imbalance is largely influenced by older age groups. Among younger professionals, such as those aged 25–29, gender distribution is nearly equal (1447 male vs. 1434 female dentists), indicating a generational shift.

Across Europe, countries such as Spain and Germany report a predominance of female dentists within the profession [7]. A similar pattern is observed in North America, including the United States and Canada, where women also represent a substantial proportion of practising dentists [8].

Nevertheless, this growing presence has not translated into equal representation at scientific meetings. Analyses of dental conferences in the UK [9] and Brazil [10] highlight persistent underrepresentation of female invited speakers. In Italy, a recent cross‐sectional study reported a marked gender gap in CME‐accredited dental conferences and among institutional leaders [11].

Within the Italian Orthodontic Society (SIDO), this contrast is particularly evident. As of 2024, female members outnumber their male counterparts (3189 vs. 2265). Nevertheless, since the first female president in 2010, only six women have served in that role. These observations provide relevant contextual background for examining gender representation among invited speakers at SIDO conferences.

Recent studies have demonstrated persistent gender disparities in orthodontic society conferences [2, 12], mirroring those observed in orthodontic research, where female authors remain underrepresented in leading and senior positions [13]. A recent study published in the European Journal of Orthodontics investigated the gender imbalance among invited speakers at the European Orthodontic Society (EOS) conferences from 2015 to 2020, revealing a consistent gap among invited speakers at EOS annual conferences [12]. Similarly, a recent article in the *American Journal of Orthodontics and Dentofacial Orthopedics* explored gender‐based disparities in the selection of invited speakers at the American Association of Orthodontists (AAO) Annual Session [8]. The study reported that, although the proportion of female members within the AAO has steadily increased, female representation among speakers remains disproportionately low compared to the percentage of female members and faculty [8].

In view of recent efforts to investigate gender disparities in conference participation within dentistry and orthodontics, and considering the limited data available for the Italian context, this cross‐sectional study aims to evaluate gender representation among invited speakers at the Italian Orthodontic Society (SIDO) congresses held from 2013 to 2023. The null hypothesis was that there is no difference in gender representation among invited speakers over the study period.

## 2 Materials and Methods

### 2.1 Data Collection

The abstract books and programmes of the SIDO congresses, including both *Spring* and *Winter* meetings held between 2013 and 2023, were reviewed to identify the gender of the invited speakers in the main sessions of each congress. For programmes that were not available electronically, specific requests were made to the SIDO scientific secretariat. Despite the cancellation of the spring meeting in 2020 due to the COVID‐19 pandemic, data from the *Winter* meeting, which was conducted online, were included in the analysis. The time span from 2013 to 2023 was selected to ensure a consistent and complete dataset, based on the availability of official conference programmes. This timeframe also allowed for the assessment of long‐term trends in gender representation and was consistent with methodological approaches adopted in similar studies on scientific conferences [14, 15].

### 2.2 Inclusion and Exclusion Criteria

Presentations delivered by invited speakers during the main sessions of the congress, lasting at least 30 min and featuring no more than three speakers, were included in the study. Only orthodontic‐related presentations from the official congress programmes were considered.

Exclusion criteria included presentations in the editor's forum, roundtable discussions, sponsored sessions, pre‐ and post‐congress sessions, collateral events, lectio magistralis and presentations by non‐SIDO societies. Additionally, non‐orthodontic presentations, such as those by dental hygienists or dental technicians, were excluded.

### 2.3 Data Extraction and Speaker Identification

Invited speakers were identified by reviewing the conference programmes. For each speaker, the following variables were collected: name of the speaker, gender (female or male), country of origin, affiliation, H‐index, year of the conference, type of meeting and the topic of the presentation (categorised into 30 thematic areas, based on previously published topic groupings [12]). Author affiliations, country of origin and H‐index data were retrieved from the Scopus database (Elsevier BV, Amsterdam, The Netherlands), a widely used platform for the evaluation of research outputs and author‐level metrics. Manual verification was performed where necessary, using institutional websites and other academic resources. Data extraction was conducted using standardised forms developed specifically for this study. Two evaluators (AB and AA) independently reviewed a sample of 20 records, following an initial pilot of 10. Discrepancies in the extracted data regarding the invited speakers were resolved through discussion to ensure consistency.

### 2.4 Speaker Gender Determination

In line with previous literature, the term ‘gender’ is used throughout this manuscript to describe the representation of male and female speakers at academic conferences. Although the classification is based on a binary male/female distinction inferred from public information, this terminology reflects a broader perspective that considers not only biological differences (sex) but also the social and cultural dimensions that influence visibility, participation and opportunity in academic settings [16].

To determine the gender of the invited speakers, the Genderize.io API (Application Programming Interface) was used [12]. This tool, widely employed in the literature, queries a database of over 100 million names and returns a predicted gender along with a probability score. For each name, the API provides a count representing the number of times the name appears in the database and a probability for the gender prediction. Following established protocols, a minimum of 5 counts and a probability threshold greater than 0.85 were required for reliable gender classification. For speakers without institutional affiliation or ambiguous gender predictions, further verification was performed manually using web searches, including social media platforms.

### 2.5 Outcomes

The primary outcome of this study was to determine the proportion of female invited speakers at SIDO congresses between 2013 and 2023. Secondary outcomes included the analysis of gender representation trends over time and the evaluation of associations between speaker characteristics (country of origin, affiliation and H‐index) and female participation. Additional analyses were conducted to assess gender distribution across different presentation topics. The study also explored the most frequent speakers and the institutions with the highest representation, considering gender differences. The gender of the SIDO presidents during the study period was also recorded based on official public sources and reported descriptively, to provide contextual information on gender balance in leadership roles.

### 2.6 Statistical Analysis

Descriptive analyses were conducted to examine the characteristics of the SIDO conferences, including counts and percentages of male and female invited speakers. A *z*‐test for proportions was used to assess the overall difference in gender representation. Cross‐tabulations were created for variables, such as year of presentation, country, H‐index (≥ 8) and affiliation (university vs. non‐university) with the primary outcome (female speaker or not). Associations between categorical variables were assessed using chi‐square tests, while Fisher's exact test was applied where appropriate due to small cell sizes.

Univariate and multivariate logistic regression analyses were employed to evaluate the effect of predictor variables, including year of presentation, country (non‐Italian speakers), H‐index (≥ 8) and affiliation (non‐academic), on the likelihood of a speaker being female. In the multivariate model, backward elimination of non‐significant predictors was applied, using a deletion criterion of *p* > 0.10, to improve model fit and retain only significant predictors. The Hosmer–Lemeshow test was used to assess the goodness‐of‐fit for the logistic regression model.

A Mantel–Haenszel test for homogeneity of odds ratios was conducted to assess whether the trends in female representation were consistent across the years. Results were presented as absolute numbers and percentages, along with odds ratios (OR) and 95% confidence intervals (95% CI) for each predictor variable.

In addition, the association between the gender of the SIDO presidents and the percentage of female invited speakers was explored using Pearson correlation.

The predefined level of statistical significance was set at *p* < 0.05. Where applicable, the kappa statistic was calculated to assess inter‐rater agreement between the two evaluators (AB, AA) for the recorded variables. Agreement was interpreted according to standard guidelines, with values indicating the strength of agreement.

## 3 Results

Inter‐rater reliability, assessed using the kappa statistic, demonstrated strong agreement between evaluators across most variables (*κ* = 0.87), indicating a high level of consistency in the identification of speaker gender and related data. However, for the classification of presentation topics, the agreement was moderate (*κ* = 0.56), suggesting some variation in the evaluators' assessments.

**FIGURE 1:**
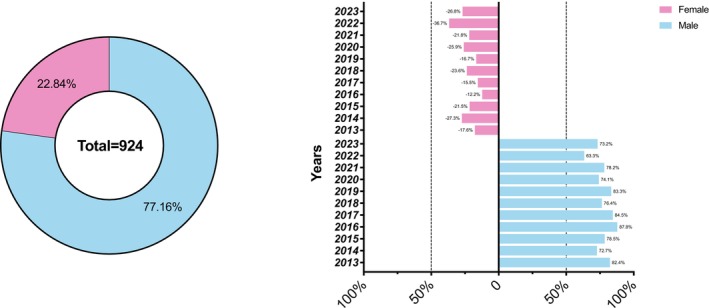
Frequency and percentage distribution of male and female invited speakers at SIDO conferences (2013–2023).

The analysis of gender representation across SIDO congresses from 2013 to 2023 revealed a significant disparity between male and female speakers (Table 1, Figure 1). Male speakers accounted for 77.2% of the total (713 male speakers), while female speakers made up only 22.8% (211 female speakers). The *z*‐test for proportions confirmed that this difference was highly statistically significant (*p* < 0.001), indicating a pronounced gender imbalance throughout the study period. A comparison between the two main annual events, the Spring and Winter meetings, revealed a statistically significant difference in female participation (*χ* ^2^ = 13.25, *p* < 0.001). Female speakers were more represented at Spring meetings (30.2% female vs. 69.8% male) than at Winter meetings (19.3% female vs. 80.7% male), indicating a greater gender imbalance during the Winter congresses.

**TABLE 1:** Logistic regression results: Distribution of male (M) and female (F) speakers at SIDO conferences from 2013 to 2023. For each year, the number (*n*) and percentage (%) of female and male speakers are presented. The totals for each year and across the entire period are included.

Year	F (*n*, %)	M (*n*, %)	Total
2013	15.0 (17.6%)	70 (82.4%)	85
2014	24.0 (27.3%)	64 (72.7%)	88
2015	14.0 (21.5%)	51 (78.5%)	65
2016	10.0 (12.2%)	72 (87.8%)	82
2017	11.0 (15.5%)	60 (84.5%)	71
2018	17.0 (23.6%)	55 (76.4%)	72
2019	15.0 (16.7%)	75 (83.3%)	90
2020	15.0 (25.9%)	43 (74.1%)	58
2021	12.0 (21.8%)	43 (78.2%)	55
2022	33.0 (36.7%)	57 (63.3%)	90
2023	45.0 (26.8%)	123 (73.2%)	168
Total	211 (22.8%)	713 (77.2%)	924

A breakdown of gender representation by year shows that the proportion of female speakers varied, with a median of 21.8% (IQR: 9.2%). The highest proportion of female speakers was in 2022 (36.7%), while the lowest was in 2016 (12.2%) (Figure 1). Despite some fluctuations, the overall trend highlights a persistent male predominance, particularly in earlier years, such as 2013 (82.4% male) and 2016 (87.8% male).

**FIGURE 2:**
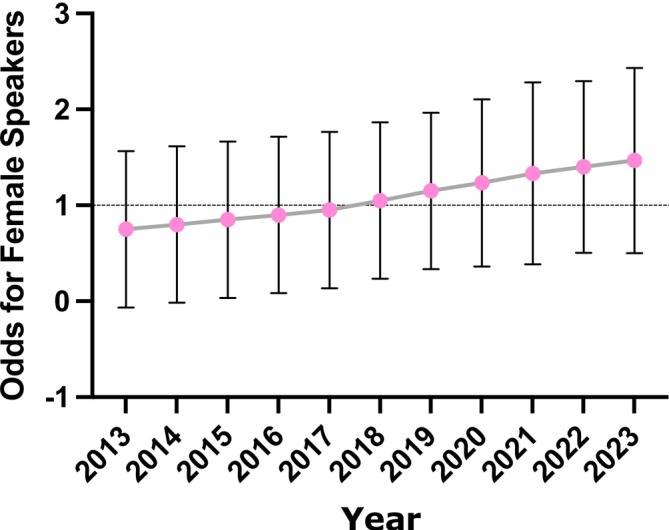
Odds ratio trends for female speakers at SIDO conferences (2013–2023).

To further explore the trend in female representation over time, a logistic regression analysis was conducted (Table 2, Figure 2). In the univariate model, the year of presentation was significantly associated with increased odds of female representation, with an odds ratio (OR) of 1.060 (95% CI [1.013–1.110], *p* = 0.011). This indicates that for each additional year, the odds of a speaker being female increased by 6%. After adjusting for other variables in the multivariate model, the year of presentation remained significant with an OR of 1.055 (95% CI [1.010–1.110], *p* = 0.015). The logistic regression analysis also indicated that event type was significantly associated with female representation. Specifically, female speakers were more likely to participate in *Spring* meetings compared to *Winter* meetings, with an odds ratio (OR) of 1.88 (95% CI [1.351–2.618], *p* < 0.001) in both univariate and multivariate models. This finding highlights that *Spring* meetings are associated with nearly twice the odds of female representation as compared to *Winter* meetings, independent of other factors. The Hosmer–Lemeshow test for model fit yielded a test statistic of 1.99 (*p* = 0.981), indicating an excellent fit for the logistic regression model. Other factors, such as country of speaker (non‐Italian speakers, OR = 1.050, *p* = 0.720), H‐index (≥ 8) (OR = 0.880, *p* = 0.402) and affiliation (speakers without affiliation, OR = 0.870, *p* = 0.360), were not significantly associated with female representation.

**TABLE 2:** Univariable and multivariable logistic regression with odds ratios (OR) and associated 95% confidence intervals (CIs) for the effect of presenter and affiliation‐related characteristics on female representation as invited speakers at the SIDO Congress (*n* = 924).

Variable	Univariable OR	95% Confidence interval	Univariable *p*‐value	Multivariable OR	95% Confidence interval	Multivariable *p*‐value
Year of presentation (increasing)	1.060	[1.013–1.110]	0.011*	1.055	[1.010–1.110]	0.015*
Country (non‐Italian speakers)	1.068	[0.779–1.465]	0.682	1.050	[0.750–1.450]	0.720
H‐Index (≥ 8)	0.873	[0.642–1.188]	0.388	0.880	[0.640–1.220]	0.402
Affiliation (speakers without affiliation)	0.857	[0.620–1.185]	0.351	0.870	[0.610–1.210]	0.360
Event type (spring meetings)	1.88	[1.351–2.618]	< 0.001*	1.88	[1.351–2.618]	< 0.001*

*Statistically significant (*p* < 0.05).

A Mantel–Haenszel test for homogeneity of odds ratios across the years was conducted to assess whether the trend of female representation was consistent over time. The test revealed a combined odds ratio of 0.33, suggesting a persistently low probability of female representation relative to male speakers. However, the *p*‐value of the Mantel–Haenszel test was 1.0, indicating no significant variation in the odds of female representation across the years, and suggesting that the increase in female speakers occurred in a homogeneous manner over the study period.

From 2013 to 2023, 4 out of the 11 presidents were women (2016, 2017, 2019 and 2021), indicating a modest increase in gender diversity at the leadership level. A Pearson correlation analysis revealed a negative association (*r* = −0.67) between having a female president and the percentage of female speakers.

The majority of speakers across the entire period were from Italy (62.3%), followed by the USA (9.6%), Spain (3.2%), Switzerland (2.9%) and Germany (2.7%).

**FIGURE 3:**
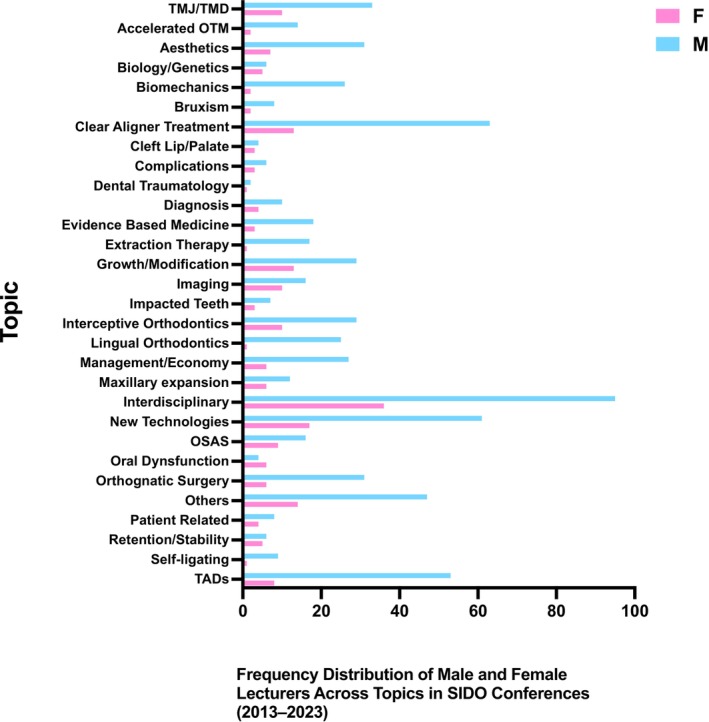
Frequency distribution of male and female lecturers across presentation topics at SIDO conferences (2013–2023).

A breakdown of the topics covered revealed that male speakers were predominant across nearly all categories. For example, in Interdisciplinary topics, 36 female speakers were compared to 95 males, while in New Technologies, there were 17 female speakers compared to 61 males. Similar patterns were observed in Clear Aligner Treatment (13 females, 63 males) and Growth/Modification (13 females, 29 males) (Figure 3).

The most frequently discussed topics during the SIDO congresses were Interdisciplinary (131 presentations), followed by New Technologies (78 presentations), Clear Aligner Treatment (76 presentations) and Temporary Anchorage Devices (TADs; 61 presentations). A deeper analysis of gender distribution across topics reveals that certain areas are predominantly male‐dominated, such as TADs, Clear Aligner Treatment and Biomechanics. In contrast, topics like Growth/Modification and Interceptive Orthodontics exhibit a more balanced gender distribution, although male speakers still lead. Interestingly, Interdisciplinary topics had higher female representation relative to others, while areas such as orthognathic surgery and self‐ligating saw minimal female participation (Figure 3).

The speakers with the most presentations during the SIDO congresses were Cocconi Renato and Nanda Ravindra S., each with 11 presentations, followed by Lombardo Luca, Caprioglio Alberto and Rosa Marco, with 10 presentations each. The most frequently invited female speaker was Chaushu Stella with seven presentations. Regarding institutional affiliations, Private Practice accounted for the largest proportion of speakers (336), followed by Università degli Studi di Ferrara (58), and Università degli Studi di Napoli Federico II [17]. Table 3 shows the top 10 most frequent speakers, whereas Table 4 lists the top 10 most represented institutions.

**TABLE 3:** Ten most frequent lecturers (invited speakers) in SIDO conferences held across 2013 and 2023.

Speaker	Number of Presentations	Gender
Cocconi Renato	11	M
Nanda Ravindra S.	11	M
Lombardo Luca	10	M
Caprioglio Alberto	10	M
Rosa Marco	10	M
Manzo Paolo	9	M
Franchi Lorenzo	8	M
Martin Domingo	7	M
Chaushu Stella	7	F
Farina Achille	7	M

**TABLE 4:** Ten most frequent affiliations of lecturers (invited speakers) in SIDO conferences held across 2013 and 2023.

Affiliation	Frequency
Private Practice	336
Università degli Studi di Ferrara	58
Università degli Studi di Napoli Federico II	29
Sapienza Università di Roma	27
Università degli Studi dell'Insubria	23
Università degli Studi di Torino	20
Università degli Studi di Firenze	18
Università degli Studi di Milano	16
University of Connecticut	16
Università degli Studi di Roma Tor Vergata	15

Finally, analysis of the H‐index revealed an upward trend in the average H‐index of speakers, peaking at 18.3 in 2019. The average H‐index of male speakers (14.52) was slightly higher than that of female speakers (12.63), indicating a slight difference in academic output between genders.

## 4 Discussion

Our study revealed a *significant gender disparity among invited speakers at SIDO congresses held between 2013 and 2023, with an average female presence of only 22.8% compared to 77.2% male speakers, despite a modest increase in female representation over the study period*. Such findings remain particularly relevant within the Italian orthodontic context and contribute to the growing body of research examining gender disparities in high‐visibility academic roles. Moreover, the timeframe covered by the study is sufficiently long to capture potential shifts related to evolving institutional practices and broader sociocultural dynamics [14, 15].

It is important to clarify that gender *equality* and gender *equity* are not synonymous. Although equality refers to equal numerical representation, equity implies the creation of fair conditions that enable all professionals, regardless of gender, to access the same opportunities for leadership and visibility [18]. This distinction is essential when interpreting the implications of our findings and understanding the systemic factors that may hinder equitable access to event participation.

Studies conducted on the AAO and the EOS confirm that gender disparity in speaker selection is a widespread issue within orthodontic conferences [2, 12]. The study on the AAO conferences found a marked underrepresentation of women as invited speakers, with women comprising only 19% of speakers, despite representing 31% of the association's active members. Interestingly, in categories involving anonymous selection, such as research awards, women represented 63% of recipients, suggesting that gender‐blind evaluation may contribute to more balanced representation. Removing identifiable information may help mitigate unintentional preferences or prejudices—such as *implicit bias*, which operates through unconscious stereotypes, and *affinity bias*, the tendency to favour individuals perceived as similar to oneself—that can affect direct invitations or selection decisions [2]. Similarly, the study on the EOS conferences found a near gender balance among speakers selected through anonymous abstract review, with women accounting for 49.0% of oral presentations between 2015 and 2020. This balance, however, did not extend to invited speakers, where only 17.9% were women, highlighting how subjectivity may influence non‐blind selection processes [12].

Our analysis revealed a notable difference in female representation between SIDO's two main annual congresses: the *Spring* meeting and the *Winter* meeting. The *Winter* meeting serves as the primary annual event, traditionally associated with higher prestige and formality. Female speakers were significantly more likely to participate at the *Spring* meetings, with 30.2% female representation compared to 19.3% at the *Winter* meetings. This finding suggests that the *Spring* meeting may provide a more favourable environment for female participation, potentially due to fewer traditional constraints, and highlights how the event setting itself may impact gender representation within the same organisation. Furthermore, the odds ratio analysis over the years reveals a gradual increase in female representation at SIDO congresses, with an estimated annual growth rate of 6% in the probability of female participation, regardless of the type of event (i.e., *Winter* or *Spring* meetings). Although this rate remains insufficient to achieve gender parity quickly, it does indicate a positive trend, suggesting that SIDO's speaker composition is gradually moving towards greater inclusivity.

As previously discussed, implicit and affinity biases are among the key mechanisms that may contribute to the underrepresentation of women, particularly when speaker selection is based on informal or non‐anonymized processes [2, 19]. However, structural factors may play an even more decisive role in shaping gender representation.

Interestingly, our analysis showed a negative association between the gender of the SIDO president and the percentage of female speakers, suggesting that the president's gender alone did not correlate with an increase in female representation among invited speakers. This finding aligns with literature suggesting that individual leadership gender may have limited impact on gender representation without accompanying structural changes [2, 18]. Although studies indicate that female involvement in organising committees or leadership positions within specialty societies can improve gender balance among speakers [2, 20], this effect is not universal and may depend on deeper organisational practices and cultural factors [12].

Beyond structural and organisational dynamics, individual‐level psychological mechanisms may also contribute to unequal participation. One such factor is the *stereotype threat*, a phenomenon in which individuals may feel pressure to conform to negative stereotypes about their capabilities in male‐dominated fields. This phenomenon has the potential to undermine self‐confidence and reduce participation in prominent roles [21]. This effect may be especially relevant at prestigious conferences where high‐profile presentations are traditionally male‐dominated, perpetuating a cycle of underrepresentation for women in such contexts.

Breaking this cycle may require proactive strategies to increase the visibility of female speakers, which can encourage participation among women attendees. Indeed, the presence of relatable role models can promote identification and engagement, as explained by *social identity theory* [22].

The analysis of the most frequent speakers at SIDO congresses confirms the overall trend of male predominance, with Renato Cocconi and Ravindra *S. Nanda* tied with 11 presentations each, followed by Luca Lombardo, Alberto Caprioglio and Marco Rosa with 10 presentations each. Stella Chaushu stands out as the only woman among the 10 most frequently invited speakers, with seven presentations, underscoring the limited female representation among the most recurrently selected speakers. This finding reflects how gender imbalance persists not only in overall participation, but also in repeated visibility opportunities typically associated with recognition and authority in the field.

The majority of invited speakers at these congresses are based in Italy, likely due to logistical and financial factors that favour national participation. Among international contributors, speakers from the United States are the most frequently represented, likely reflecting global trends in research influence across most field [23].

Given the predominance of nationally based speakers, it is particularly relevant to consider the gender composition of the dental profession in Italy. The underrepresentation of women among invited speakers may, at least in part, reflect the broader gender imbalance within the national professional landscape, where only 29.3% of dentists are female compared to 70.7% male, potentially limiting the available pool of female candidates for high‐visibility roles such as congress speakers (Supporting Information).

However, numerical imbalance alone does not fully explain the lack of female visibility. A further contributing factor may be the well‐known *leaky pipeline* phenomenon, which describes the progressive attrition of women at successive career stages, particularly in STEM and academic professions [24]. Despite ongoing progress towards gender parity in university graduation rates across many contexts, women remain underrepresented in senior roles. In Italy, only 38.7% of senior positions in STEM and related fields are held by women [25], while globally the share drops to 28.2% [26]. These disparities underscore the persistence of structural and cultural dynamics that limit women's access to influential roles. Italy ranks 102nd globally in female representation in professional leadership roles [26], further illustrating the extent of this imbalance. Such a gap between the number of women entering the profession and those reaching senior roles reflects a *glass ceiling effect*, where invisible barriers, often rooted in structural and cultural biases, limit women's advancement into high‐visibility roles [13, 27]. These barriers restrict access to leadership opportunities and create a disparity between the initial number of women in the profession and those who progress into senior positions [11, 28]. This phenomenon has been documented in various orthodontic societies, where women are frequently confined to administrative roles and remain underrepresented in leadership positions [29]. A similar trend is evident in academic publishing, particularly in key authorship positions such as first or last author [13]. In recent orthodontic publications, women represented only 34% of all listed authors, highlighting structural barriers that limit female visibility and advancement both as invited speakers and as contributors in leading academic roles [13]. A recent bibliometric analysis further supports this, showing that among the most‐cited orthodontic articles, although 67.7% included at least one female author, only 4.6% were authored exclusively by women and female researchers were markedly underrepresented in senior authorship roles [17].

An analysis of the topics covered at SIDO congresses reveals a predominance of interdisciplinary themes, reflecting a trend towards integrated treatment strategies that engage multiple dental and medical specialties alongside orthodontics [30]. Among the most frequently represented topics, ‘New Technologies’, ‘Clear Aligner Treatment’, and ‘TADs’ have emerged as areas of growing interest, in line with recent literature emphasising their central role in contemporary orthodontic practice [31, 32].

These topics are predominantly presented by male speakers, reflecting a gendered distribution of expertise that parallels patterns in the orthodontic literature. Male authors are more prevalent in technology‐oriented fields, potentially reinforcing disparities in both publishing and speaker representation [13]. Furthermore, discipline‐specific *perception biases* have been documented, with male experts generally perceived as more credible in natural sciences and female experts considered more credible in the social sciences [4]. This suggests a broader cultural bias in how expertise is evaluated across different fields. In the context of orthodontic conferences, this highlights the importance of ensuring balanced representation across both clinical and research‐focused presentations, in order to reach a broader audience and reduce perception‐based bias [3]. Conversely, topics such as interceptive orthodontics and interdisciplinary approaches show a more balanced gender distribution, consistent with findings that highlight greater female involvement in areas related to holistic care and psychosocial dimensions of treatment [13].

In addition to thematic preferences and institutional affiliations, academic productivity was also evaluated as a potential factor influencing speaker selection. It is also worth noting that a substantial proportion of invited speakers lacked formal academic affiliations, reflecting the fact that many experienced clinicians in dentistry work predominantly in private practice rather than academic institutions [31]. The H‐index, a widely used indicator of scholarly output and impact, was analysed to determine whether differences in academic profiles could help explain the observed gender disparities. The analysis revealed an upward trend in H‐index values, peaking at an average of 18.3 in 2019. This increase suggests a growing emphasis on scientific productivity among invited speakers, reflecting SIDO's interest in selecting professionals with demonstrated academic impact. The H‐index is commonly used to assess both the quantity and quality of an author's publications, making it a critical measure of scientific influence [31]. The relatively small difference in average H‐index between male (14.52) and female (12.63) speakers suggests that both genders exhibit comparable scientific profiles. This further reinforces that the underrepresentation of women among invited speakers is unlikely to be explained by differences in scholarly productivity.

Overall, the findings highlight the persistence of gender disparities in high‐visibility scientific roles, despite comparable academic profiles across genders. Promoting fair and inclusive participation remains a key challenge for scientific societies seeking to reflect the diversity of the professional community.

## 5 Limitations and Future Directions

Several limitations should be acknowledged in this study. This analysis relied on historical data obtained from public sources and congress records, which may contain inaccuracies or reflect yearly variations that do not fully represent the gender composition. Additionally, historical data may not account for recent initiatives by scientific societies aimed at enhancing gender representation, potentially underestimating recent improvements. Furthermore, the exclusive focus on a single national orthodontic society may limit the generalizability of the findings, as observed trends might not fully represent the broader national or international orthodontic landscape. The use of the H‐index as a measure of scientific impact is also limiting, as it may undervalue professionals predominantly engaged in clinical practice, whose expertise and influence might not be fully captured by scholarly output alone. Additionally, while the study provides robust quantitative data, it does not explore the organisational or cultural factors that may influence equitable access to speaking opportunities [18, 33].

Future research could examine the gender composition of speaker selection committees, assess the impact of gender‐balanced program panels and conduct comparative studies across national and international orthodontic societies. Tracking initiatives such as speaker training programmes, mentorship and institutional policies may offer further insights into strategies that promote equity [18, 33].

Scientific societies can play a key role by adopting transparent selection criteria, ensuring gender‐balanced organising groups and systematically monitoring representation metrics [14]. Defining and publicly sharing clear speaker selection guidelines may serve as a structural safeguard against gender‐blindness, helping to ensure that gender balance is actively considered throughout the decision‐making process [29, 33]. By fostering inclusive environments, adopting evidence‐based procedures and supporting continuous monitoring, scientific societies are well positioned to lead such efforts [34]. Increasing the visibility of female speakers not only promotes inclusion but also provides role models that inspire younger generations and reinforce trust in scientific leadership [34, 35].

## 6 Conclusions

The findings of this study reveal a significant gender disparity among invited speakers at SIDO congresses from 2013 to 2023. Although there has been a modest annual increase in female representation, gender parity remains a distant goal. Furthermore, the higher representation of female speakers at *Spring* meetings suggests that event type and perceived prestige may influence speaker diversity. Taken together, the evidence gathered from the present study suggests the following:

- Female representation remained below 25%, with an estimated 6% annual increase in participation likelihood.
- *Spring* meetings consistently featured a higher proportion of female speakers than *Winter* meetings, suggesting that event format may influence speaker diversity.
- The 10 most frequently invited speakers included only one woman, indicating persistent disparities in visibility across multiple years.
- No positive correlation was observed between female presidency and female speaker representation, implying that broader structural dynamics may play a more significant role.

These findings reinforce the relevance of transparent, gender‐conscious selection strategies, which may help foster inclusivity in orthodontic conferences. By documenting existing disparities, this study provides a foundation for future actions aimed at improving gender equity within SIDO and potentially informing policies across other scientific societies.

## Author Contributions

Conceived and designed the study: A.B.; acquisition, analysis or interpretation: A.B.; drafting the work: A.B. and A.A.; data collection: E.K. and A.B.; wrote the article: A.B. and A.A.; critical revision of the article: C.M.; final approval of the article: V.L. and A.U.; response to reviewers and manuscript revision: A.B.

## Ethics Statement

The authors have nothing to report.

## Consent

The authors have nothing to report.

## Conflicts of Interest

The authors declare no conflicts of interest.

## Supporting information


Data S1

## Data Availability

The data supporting the findings of this study are available from the corresponding author upon reasonable request.
